# Association of pre- and early-pregnancy factors with the risk for gestational diabetes mellitus in a large Chinese population

**DOI:** 10.1038/s41598-021-86818-7

**Published:** 2021-04-01

**Authors:** Min Zhao, Shuyu Yang, Tzu Chieh Hung, Wenjie Zheng, Xiaojie Su

**Affiliations:** 1 Department of Gynecology and Obstetrics, The First Affiliated Hospital of Xiamen University, No. 55, Zhenhai Road, Siming District, Xiamen, 361003 Fujian China; 2 Computer Management Center, The First Affiliated Hospital of Xiamen University, Xiamen, Fujian China; 3 National Institute for Data Science in Health and Medicine, Xiamen University, Xiamen, Fujian China

**Keywords:** Gestational diabetes, Risk factors

## Abstract

Gestational diabetes mellitus (GDM) has aroused wide public concern, as it affects approximately 1.8–25.1% of pregnancies worldwide. This study aimed to examine the association of pre-pregnancy demographic parameters and early-pregnancy laboratory biomarkers with later GDM risk, and further to establish a nomogram prediction model. This study is based on the big obstetric data from 10 “AAA” hospitals in Xiamen. GDM was diagnosed according to the International Association of Diabetes and Pregnancy Study Group (IADPSG) criteria. Data are analyzed using Stata (v14.1) and R (v3.5.2). Total 187,432 gestational women free of pre-pregnancy diabetes mellitus were eligible for analysis, including 49,611 women with GDM and 137,821 women without GDM. Irrespective of confounding adjustment, eight independent factors were consistently and significantly associated with GDM, including pre-pregnancy body mass index (BMI), pre-pregnancy intake of folic acid, white cell count, platelet count, alanine transaminase, albumin, direct bilirubin, and creatinine (*p* < 0.001). Notably, per 3 kg/m^2^ increment in pre-pregnancy BMI was associated with 22% increased risk [adjusted odds ratio (OR) 1.22, 95% confidence interval (CI) 1.21–1.24, *p* < 0.001], and pre-pregnancy intake of folic acid can reduce GDM risk by 27% (adjusted OR 0.73, 95% CI 0.69–0.79, *p* < 0.001). The eight significant factors exhibited decent prediction performance as reflected by calibration and discrimination statistics and decision curve analysis. To enhance clinical application, a nomogram model was established by incorporating age and above eight factors, and importantly this model had a prediction accuracy of 87%. Taken together, eight independent pre-/early-pregnancy predictors were identified in significant association with later GDM risk, and importantly a nomogram modeling these predictors has over 85% accuracy in early detecting pregnant women who will progress to GDM later.

## Introduction

As a serious complication of pregnancy, gestational diabetes mellitus (GDM) is a major public health problem^1^, affecting approximately 1.8–25.1% of pregnancies worldwide^2^. This problem has attracted much attention in obstetrics, because GDM usually leads to a variety of adverse maternal and neonatal outcomes, including cesarean delivery, fetal macrosomia, preterm birth, preeclampsia, and neuropsychiatric morbidity^3–5^. Despite a vast amount of resources spent and years of progress made in basic and clinical research^6,7^, challenges remain in the identification of pregnant women who are at a high risk of developing GDM in the second or third trimester and who could benefit from effective prevention or timely intervention strategies.

Several risk factors such as older age and pre-pregnancy obesity^8^ have been well established in susceptibility to GDM, yet the prediction accuracy is not ideal^9,10^, mainly because the development of GDM is a multifactorial and complex process, involving many risk factors^11^. Considering that glucose challenge test and oral glucose tolerance test (OGTT) for the diagnosis of GDM are usually performed during the third trimester (24th to 28th weeks), it is of importance to seek promising risk predictors especially at early pregnancy (8th to 12th weeks of pregnancy)^12^ to identify high-risk women for effective surveillance and prevention efforts, which can gain 12 to 16 weeks of intervention time. Currently, published data on this subject mainly focus on demographic parameters. In addition, considering the complex nature of GDM, the impact of any risk predictor on the development of GDM may be small when assessed in isolation, but may be more obvious in combination with other risk factors^13,14^. In the literature, dozens of studies have attempted to construct a risk prediction model for GDM^14–16^, yet the prediction performance remains untested or less satisfactory, curbing its translation into clinical application.

To fill this gap in knowledge and yield more information for future research, we, based on the big obstetric data from Xiamen, China, aimed to examine the association of potential risk predictors (including pre-pregnancy demographic parameters and early-pregnancy laboratory biomarkers) with later GDM risk, and further to establish a nomogram prediction model by regressing conventionally-recognized and newly-identified predictors of significance.

## Methods

### Study design and ethical approval

This is a multicenter hospital-based cohort study. Maternal and child health data from 10 “AAA” hospitals in Xiamen during the period between 2008 and 2018 were obtained from the Xiamen Primary Health Information System. The 10 “AAA” hospitals included The First Affiliated Hospital of Xiamen University, The First Affiliated Hospital of Xiamen University—Siming Branch, The First Affiliated Hospital of Xiamen University—Xinglin Branch, Zhongshan Hospital Xiamen University, The Second Affiliated Hospital of Xiamen Medical College, The First Affiliated Hospital of Xiamen University—Tongmin Branch, Xiamen Maternal and Child Care Service Center, Xiamen Haicang Hospital, Xiamen Xianyue Hospital, and Xiamen Traditional Chinese Medicine Hospital. Informed consent was signed by all participants from each participating hospital.

Ethical approval was obtained from the Institutional Review Boards at all participating hospitals, and informed consent was signed from all participants undergoing direct interview. Data sharing certification complied with the relevant policies set forth by the Xiamen Health Bureau.

### Study participants

Study participants were restricted to gestational women aged ≥ 18 years who had the expected date of confinement falling from the year 2008 to 2018, as well as data on standard glucose challenge test and/or OGTT. Gestational women with pre-pregnancy diabetes mellitus were excluded from the current analysis.

### Diagnosis of gestational diabetes mellitus

GDM was diagnosed according to criteria set forth by the International Association of Diabetes and Pregnancy Study Group (IADPSG)^17^. During the 24th to 28th gestational weeks, women who had non-fasting plasma glucose ≥ 7.8 mmol/L with a 1-h 50-g glucose challenge test were requested to undertake a 2-h 75-g OGTT, which was carried out in the morning after an overnight fasting of over 8 h, with blood samples abstracted at fasting, 1 h and 2 h after the glucose load. A pregnant woman is diagnosed to have GDM if one or more of the following criteria are satisfied: fasting plasma glucose ≥ 5.1 mmol/L, 1-h plasma glucose ≥ 10.0 mmol/L, or 2-h plasma glucose ≥ 8.5 mmol/L.

### Demographic characteristics

Pre-pregnancy demographic data were self-reported by study participants at the first pre-natal visit during the 8th to 12th weeks of pregnancy, including age, age at menarche, cigarette smoking, alcohol drinking, education, medical histories of diabetes mellitus and hypertension, pre-pregnancy intake of folic acid, pregnancy week, the presence of hemopathy, epilepsy, hyperthyroidism, cardiovascular diseases, liver diseases, kidney diseases, and lung diseases, as well as maternal family histories of diabetes mellitus and hypertension. Data on the previous histories of GDM and macrosomia were missing.

Cigarette smoking status was classified as never smoking and ever (former or current) smoking. Alcohol drinking was classified as never drinking and ever (former or current) drinking. Education was classified as high (college or equivalent degree or above) and low (high school degree or below) education. A maternal family history of diabetes mellitus or hypertension was defined as one or more of affected relatives within three generations who had clinically confirmed diabetes mellitus or hypertension.

Body height (to the nearest 0.1 cm) and pre-pregnancy weight (to the nearest 0.1 kg) were measured by nurses or trained staff. Pre-pregnancy body mass index (BMI) was calculated as pre-pregnancy weigh (kg) divided by the square of body height (m).

### Laboratory biomarkers

Besides recording demographic information, fasting blood samples were also abstracted at the first pre-natal visit during the 8th to 12th weeks of pregnancy for the measurement of laboratory biomarkers. In this study, because coverage on laboratory biomarkers differed across participating hospitals, only white cell count, platelet count, hemoglobin, alanine transaminase (ALT), aspartate aminotransferase (AST), albumin, direct bilirubin, conjugated bilirubin, creatinine, and blood urea nitrogen (BUN) were included for analysis. In view the strong biological relevance between direct bilirubin and conjugated bilirubin, only direct bilirubin was retained in the analysis. The concentrations of these biomarkers were quantified by the clinical laboratory or department of each participating hospital.

### Statistical analyses

Utilizing the extract-transform-load process in SQL server 2008 R2, crude variables containing missing (> 50%) values were removed. In addition, implausible values or extreme outliers that might represent transcription or data entry errors were checked. All outliers were reported to the data entry technicians, who corrected the database by comparing against the paper records or in consultation with the obstetricians. Then, variables were imported into the Stata software version 14.1 for Windows (Stata Corp, TX) for data cleaning and management.

All study participants were divided into the patient group and the control group according to the diagnosis of GDM. The distributions of continuous characteristics, summarized as mean (standard deviation) and median (interquartile range), were appraised for normality by use of 1-sample Kolmogorov–Smirnov test. Continuous characteristics that were found to deviate from normality were compared between the two groups using the Wilcoxon–Mann–Whitney rank sum test, and the t test otherwise. Categorical characteristics, summarized as count and percentage, were compared using the χ^2^ test.

To assess the possibility of non-random measurement error for clinical biomarkers resulting from procedural differences across the ten “AAA” hospitals in this study, the intraclass correlation coefficient (ICC) was employed, and it is a statistic that can be used to quantify the degree to which observations within a cluster differ from those between clusters^18^. The confidence limits for ICC were estimated using the multivariable delta method^19^. The ICC ranges from 0 to 1, with an ICC of 0 indicating the variance in clinical biomarkers is not due to variation between the hospitals.

The identification of significant factors for the risk of GDM was done by using the Logistic regression analysis before (model 0) and after adjusting for confounding factors (model 1 and model 2). Confounders in model 1 included age, cigarette smoking, alcohol drinking, education, and age at menarche, and confounders in model 2 additionally included maternal family histories of diabetes mellitus and hypertension, and the presence of hemopathy, epilepsy, hyperthyroidism, cardiovascular diseases, liver diseases, kidney diseases, and lung diseases. The risk for GDM was denoted by odds ratio and its 95% confidence interval (95% CI). Significant factor is identified if statistical significance (*p* value < 0.05) is fulfilled simultaneously across three different models.

For continuous significant factors identified, Spearman rank correlation coefficients were calculated to check for collinearity. If pairwise correlation coefficient is over 0.6, only one factor is retained for analysis.

To examine the prediction performance of significant independent factors, two models were constructed: basic model and full model. Factors in the basic model included age, alcohol drinking, cigarette smoking, education, age at menarche, maternal family histories of diabetes mellitus and hypertension, as well as the presence of hemopathy, epilepsy, hyperthyroidism, cardiovascular diseases, liver diseases, kidney diseases, and lung diseases. The full model additionally included significant independent factors. Prediction accuracy gained by adding significant independent factors to the basic model was appraised by use of the following statistics or tests: Akaike information criteria (AIC), Bayesian information criteria (BIC)^20^, likelihood ratio test, net reclassification improvement (NRI), integrated discrimination improvement (IDI)^21^, and area under receiver operating characteristic curve (AUROC) under both calibration and discrimination aspects. What’s more, the net benefits gained by adding significant independent factors were visually appraised in decision curve analysis^22^. In the plot of decision curve analysis, the X-axis represents thresholds for GDM risk, and the Y-axis represents net benefits hinged on different thresholds. The farthest the curve is, the highest the net benefit is.

To facilitate clinical application, a risk prediction model illustrated as a nomogram was established by regressing conventionally-recognized and newly-identified significant independent factors. The performance of this nomogram model was appraised by using both concordance index (C-index, which equals to the AUROC) and calibration curve. The larger the C-index, the more accurate was the risk prediction for GDM. The C-index ranges from 0.0 to 1.0, and it is generally accepted that the C-index of < 0.7 suggests no improvement in model performance^23^. In calibration curve, the 45° line denotes the optimal prediction in calibration curve, showing how far the predicted probabilities of the nomogram are from the actual observations. The nomogram model was established using the R programming environment (version 3.5.2) “rms” package^24^.

All reported *p* values are based on two-sided tests of significance, and *p* value less than was considered as statistically significant.

## Results

### Study participants

Data on 258,466 gestational women at 10 “AAA” hospitals were extracted from the Xiamen Primary Health Information System. After excluding 64,335 women with missing values on glucose challenge test and/or OGTT, 3161 women with abnormal values, and 578 women with pre-pregnancy diabetes mellitus, 187,432 gestational women were eligible for inclusion, with 49,611 women diagnosed with GDM and 137,821 women free of GDM in the final analysis.

### Baseline characteristics

Table 1 shows the baseline characteristics of all study participants. Women with GDM were older (mean: 29.33 vs. 28.34 years, *p* < 0.001), had higher pre-pregnancy BMI (mean: 21.27 vs. 20.59 kg/m^2^, *p* < 0.001) and lower education levels (23.20% vs. 25.70%, *p* < 0.001) than women free of GDM. No differences were noted for age at menarche, cigarette smoking, alcohol drinking, and maternal family history of hypertension between the two groups.

**Table 1:** The baseline characteristics of the study participants.

Characteristics	Patients (n = 49,611)	Controls (n = 137,821)	p*
**During the 8th to 12th gestational weeks**			
Age (years)	29.33 (4.88)	28.34 (4.45)	< 0.001
Pre-pregnancy body mass index (kg/m^2^)	20.81 (19.10–23.01)	20.13 (18.73–22.04)	< 0.001
Age at menarche (years)	14 (13–15)	14 (13–15)	0.049
Alcohol drinking	20 (0.04%)	72 (0.05%)	0.294
Cigarette smoking	8 (0.02%)	33 (0.02%)	0.306
High education	11,771 (23.73%)	36,067 (26.17%)	< 0.001
Maternal family history of diabetes mellitus	1790 (3.61%)	3498 (2.54%)	< 0.001
Maternal family history of hypertension	3200 (6.45%)	8725 (6.33%)	0.496
Hypertension	120 (0.24%)	224 (0.16%)	< 0.001
Pre-pregnancy intake of folic acid	49,444 (99.66%)	137,494 (99.76%)	< 0.001
Hemopathy	110 (0.22%)	319 (0.23%)	0.663
Epilepsy	17 (0.03%)	58 (0.04%)	0.444
Hyperthyroidism	446 (0.9%)	1306 (0.95%)	0.29
Cardiovascular diseases	125 (0.25%)	304 (0.22%)	0.228
Liver diseases	2468 (4.97%)	6903 (5.01%)	0.603
Kidney diseases	153 (0.31%)	524 (0.38%)	0.019
Lung diseases	103 (0.21%)	283 (0.21%)	0.959
Hemoglobin (g/L)	124 (117–131)	124 (117–130)	0.477
White cell count (10^9^/L)	8.50 (7.26–9.91)	8.11 (6.96–9.48)	< 0.001
Platelet count (10^9^/L)	229 (196–265)	225 (194–261)	< 0.001
Alanine transaminase (U/L)	14 (10.90–20)	14 (10.70–19.50)	< 0.001
Aspartate aminotransferase (U/L)	16.20 (14–20)	16.10 (14–20)	0.282
Albumin (g/L)	42 (39.10–44.40)	42.50 (40–44.70)	< 0.001
Direct bilirubin (μmol/L)	9.50 (7.40–12.10)	9.80 (7.60–12.50)	< 0.001
Creatinine (μmol/L)	53 (45–63)	53 (45–64)	< 0.001
Blood urea nitrogen (mmol/L)	2.80 (2.30–3.37)	2.80 (2.30–3.34)	0.237
**During the 24th to 28th gestational weeks**			
OGTT fasting glucose (mmol/L)	5.20 (4.69–6.54)	4.42 (4.18–4.66)	< 0.001
OGTT1h glucose (mmol/L)	9.270 (7.77–10.40)	7.48 (6.43–8.42)	< 0.001
OGTT2h glucose (mmol/L)	7.80 (6.51–8.90)	6.320 (5.60–7.06)	< 0.001

*SD* standard deviation, *IQR* inter-quartile range (25% quantile to 75% quantile), *OGTT* oral glucose tolerance test. Besides age expressed as mean (SD), the other continuous variables are expressed as median (IQR). Categorical data are summarized as count (percentage).

*Between patients and controls, age was compared by using the t test, and the other continuous variables were compared using the Wilcoxon–Mann–Whitney rank sum test; all categorical variables were compared using the χ^2^ test.

The possibility of measurement error for clinical biomarkers resulting from procedural differences across multiple hospitals was assessed using the ICC statistic (Supplementary Table 1). The ICCs for all clinical biomarkers were all relatively low (< 0.07), indicating a low probability of clustering within hospitals and a less likelihood of differences in measurement techniques between hospitals.

### Identification of significant factors

Three models, namely model 0, model 1, and model 2, were constructed under the Logistic regression models to identify potential factors in significant association with GDM risk (Table 2). Before and after adjusting for confounders, eight factors were consistently and significantly associated with GDM at a significance level of 0.001, including pre-pregnancy BMI, pre-pregnancy intake of folic acid, white cell count, platelet count, alanine transaminase, albumin, direct bilirubin, and creatinine. Of note, per 3 kg/m^2^ increment in pre-pregnancy BMI was associated with a 22% increased risk of GDM (adjusted OR 1.22, 95% CI 1.21–1.24, *p* < 0.001), and pre-pregnancy intake of folic acid can reduce the risk by 27% (adjusted OR: 0.73, 95% CI 0.69–0.79, *p* < 0.001). Additionally, increased levels of white cell count, platelet count, and alanine transaminase were associated with a significantly increased risk of GDM, whereas that of albumin, direct bilirubin, and creatinine corresponded to a reduced risk.

**Table 2:** Identification of significant pre- and early-pregnancy factors for later gestational diabetes mellitus before and after adjusting for confounding factors.

Characteristics	Model 0			Model 1			Model 2		
	OR	95% CI	*p*	OR	95% CI	*p*	OR	95% CI	*p*
Pre-pregnancy BMI (per + 3 kg/m^2^)	1.28	1.26–1.29	< 0.001	1.23	1.21–1.24	< 0.001	1.22	1.21–1.24	< 0.001
Pre-pregnancy intake of folic acid (yes vs. no)	0.76	0.71–0.81	< 0.001	0.74	0.69–0.79	< 0.001	0.73	0.69–0.79	< 0.001
Hypertension (+ vs. −)	1.48	1.19–1.85	0.001	1.28	1.00–1.58	0.046	1.26	0.99–1.57	0.062
Hemopathy (+ vs. −)	0.95	0.77–1.18	0.663	0.95	0.76–1.18	0.634	0.95	0.76–1.18	0.614
Epilepsy (+ vs. −)	0.81	0.47–1.39	0.445	0.85	0.49–1.46	0.551	0.86	0.5–1.48	0.577
Hemoglobin (per + 40 g/L)	1.02	0.98–1.06	0.372	1.01	0.98–1.06	0.474	1.01	0.98–1.06	0.467
White cell count (per + 1 * 10^9^)	1.10	1.1–1.11	< 0.001	1.11	1.1–1.11	< 0.001	1.11	1.10–1.11	< 0.001
Platelet count (per + 50 * 10^9^)	1.06	1.05–1.07	< 0.001	1.06	1.05–1.07	< 0.001	1.06	1.05–1.07	< 0.001
Alanine transaminase (per + 20 U/L)	1.06	1.04–1.08	< 0.001	1.05	1.03–1.06	< 0.001	1.05	1.03–1.06	< 0.001
Aspartate aminotransferase (per + 20 U/L)	1.04	1.01–1.07	0.004	1.03	1.00–1.06	0.057	1.03	1.00–1.06	0.058
Albumin (per + 5 g/L)	0.85	0.84–0.86	< 0.001	0.86	0.85–0.88	< 0.001	0.86	0.85–0.87	< 0.001
Direct bilirubin (per + 5 μmol/L)	0.93	0.93–0.95	< 0.001	0.93	0.92–0.95	< 0.001	0.94	0.92–0.95	< 0.001
Creatinine (per + 15 μmol/L)	0.96	0.95–0.97	< 0.001	0.95	0.94–0.96	< 0.001	0.95	0.84–0.96	< 0.001
Blood urea nitrogen (per + 10 mmol/L)	1.02	0.94–1.10	0.708	1.03	0.95–1.12	0.456	1.02	0.94–1.11	0.560

*OR* odds ratio, *95% CI* 95% confidence interval, *BMI* body mass index. No confounders were adjusted in model 0; variables under adjustment in model 1 included age, alcohol drinking, cigarette smoking, education, and age at menarche; additional variables under adjustment in model 2 included maternal family histories of diabetes mellitus and hypertension, and the presence of hemopathy, epilepsy, hyperthyroidism, cardiovascular diseases, liver diseases, kidney diseases, and lung diseases on the basis of model 1.

### Correlation analysis of significant factors

Spearman correlation analysis was performed to test collinearity of significant continuous factors identified above. As reflected by the Spearman correlation coefficients (Table 3). The correlation coefficients ranged from − 0.08 to 0.29.

**Table 3:** Correlation analysis of continuous significant factors in predicting gestational diabetes mellitus in both patients and controls.

Correlation Coef	Pre-pregnancy BMI	White cell count	Platelet count	ALT	Albumin	Creatinine	Direct bilirubin
Pre-pregnancy BMI	1.000	0.120	0.162	0.081	− 0.021	0.044	− 0.070
White cell count	0.146	1.000	0.290	0.065	− 0.073	0.009	− 0.076
Platelet count	0.195	0.276	1.000	0.033	0.054	− 0.038	− 0.049
ALT	0.104	0.073	0.062	1.000	0.027	0.005	0.005
Albumin	0.017	− 0.075	0.092	0.075	1.000	0.109	0.154
Creatinine	0.046	0.000	− 0.029	0.015	0.133	1.000	0.001
Direct bilirubin	− 0.056	− 0.084	− 0.050	0.015	0.147	0.014	1.000

*coef.* Coefficient, *ALT* alanine transaminase. The lower triangular data represent the correlation coefficients in patients, and the upper triangular data represent the correlation coefficients in controls.

### Prediction performance assessment

The prediction performance of eight significant independent factors was assessed by means of calibration and discrimination statistics. As showed in Table 4, the differences in AIC and BIC values were significantly greater than 10 between the basic model and the full model, indicating the significant prediction by adding eight significant factors, which was further confirmed by the likelihood ratio test (*p* < 0.001).

**Table 4:** Calibration and discrimination statistics for the addition of eight significant pre- and early-pregnancy factors identified to the basic model.

Statistics	Basic model	Full model
**Calibration**		
Akaike information criterion (AIC)	215,380	212,349
Bayesian information criteria (BIC)	215,501	212,586
Likelihood ratio (LR) test	*p* < 0.001	
**Discrimination**		
Net reclassification improvement (NRI)	*p* < 0.001	
Integrated discrimination improvement (IDI)	*p* < 0.001	
Area under receiver operating characteristic curve (AUROC)	*p* < 0.001	

Variables in the basic model included age, alcohol drinking, cigarette smoking, education, age at menarche, maternal family histories of diabetes mellitus and hypertension, as well as the presence of hemopathy, epilepsy, hyperthyroidism, cardiovascular diseases, liver diseases, kidney diseases, and lung diseases. Variables in the full model additionally included pre-pregnancy body mass index, pre-pregnancy intake of folic acid, hypertension, while cell count, platelet count, alanine transaminase, albumin, direct bilirubin, and creatinine.

In addition, the significance of NRI and IDI statistics revealed that the addition of eight significant independent factors to the basic model can differentiate women with GDM from gestational women under study, which was further reinforced by the significant AUROC difference between the two models (*p* < 0.001).

**Figure 1:**
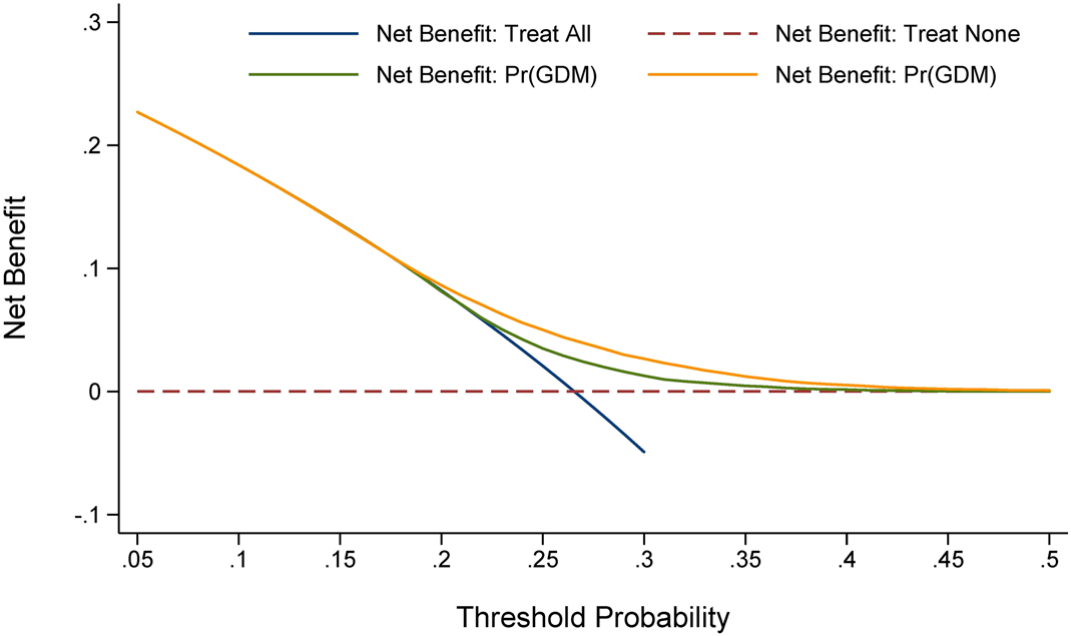
Decision curve analysis of eight pre- and early-pregnancy significant independent factors in predicting gestational diabetes mellitus later. *GDM* gestational diabetes mellitus. The orange solid line corresponds to the basic model that includes age, alcohol drinking, cigarette smoking, education, age at menarche, maternal family histories of diabetes mellitus and hypertension, as well as the presence of hemopathy, epilepsy, hyperthyroidism, cardiovascular diseases, liver diseases, kidney diseases, and lung diseases. The green solid line corresponds to the full model that includes both factors in the basic model and the eight newly-identified unrelated significant factors, including pre-pregnancy body mass index, pre-pregnancy intake of folic acid, white cell count, platelet count, alanine transaminase, albumin, direct bilirubin, and creatinine. Over threshold probabilities of 0.2, the net benefit gained by adding the eight significant factors was greater than that in the basic model.

Furthermore in the decision curve analysis, there were evident net benefits after adding these eight factors to the basic model (Fig. 1).

### Establishment of a risk prediction model

**Figure 2:**
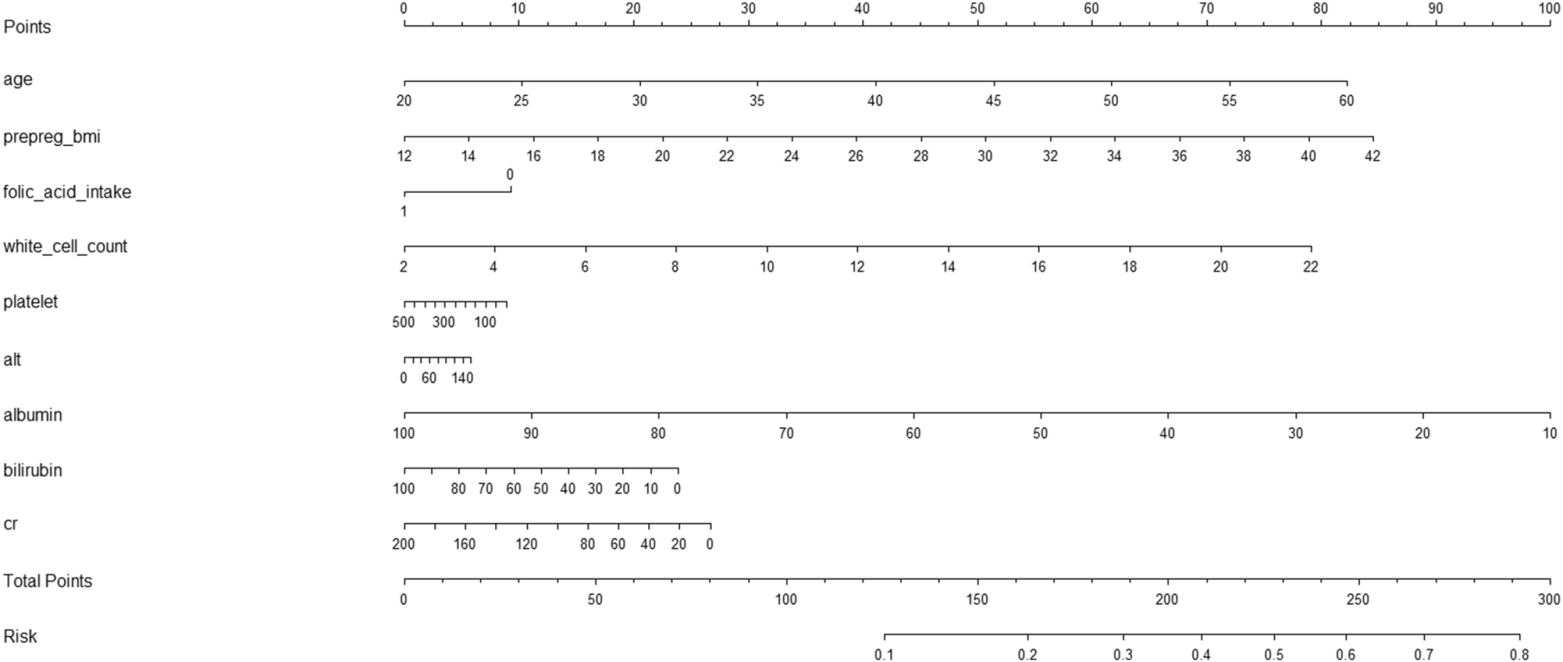
Establishment of a risk prediction nomogram based on pre- and early-pregnancy significant independent factors for gestational diabetes mellitus later. *BMI* body mass index, *ALT* alanine transaminase, *Cr* creatinine, *GDM* gestational diabetes mellitus. This nomogram can be used to manually obtain predicted values from a regression model that was fitted with the pre- and early-pregnancy significant independent factors. In detail, there is a reference line at the top for reading scoring points (range 0–100) from all factors in the regression model, which were summed together to calculate the total points, and then the predicted values can be read at the bottom.

In view of the nonlinear relationship between continuous significant factors and the decent prediction performance, a risk prediction model was hence established by using the nomogram technique by modeling age and the eight identified factors of significance, including pre-pregnancy BMI, pre-pregnancy intake of folic acid, white cell count, platelet count, alanine transaminase, albumin, direct bilirubin, and creatinine, as illustrated in Fig. 2.

This nomogram model had a good prediction accuracy, with the C-index of being 87%, indicating 87% correct model identification of the high-risk women who will experience GDM across all possible pairs of pregnant women. The calibration curve for this nomogram model is presented in Supplementary Figure 1. In addition, positive predictive value and negative predictive value for differentiating the presence and absence of GDM were estimated to be 72.91% and 93.69%, respectively.

## Discussion

The aim of this study was to examine the association of promising pre-pregnancy demographic parameters and early-pregnancy laboratory biomarkers with the later risk of GDM, and further to establish a prediction model. The key findings of our analysis were the identification of eight independent pre-/early-pregnancy predictors in significant association with the later risk of GDM, and importantly incorporation of these significant predictors in a nomogram model had over 85% accuracy in early detecting pregnant women who will progress to GDM at the third trimester.

A growing number of epidemiological parameters and laboratory biomarkers have been evaluated in prediction of GDM in the medical literature. For instance, Guo and colleagues retrospectively analyzed 3956 Chinese women who underwent their first antenatal visits, and found that age, pre-pregnancy obesity, first-trimester, fasting plasma glucose, and a family history of diabetes mellitus were significant predictors of later GDM^16^. In addition, many laboratory biomarkers in circulation such as fibroblast growth factors^25^, fatty acids^26^, and ferritin^27^ have been listed as promising drivers of GDM. Currently, one of the greatest challenges facing global obstetricians is the identification of proper early-pregnancy laboratory biomarkers and the establishment of a prediction model incorporating some well-established factors for GDM, yet for a few established risk factors such as age and obesity, the results of most studies are not often reproducible for other parameters or biomarkers. The reasons for the repeated failure are not fully understood, and may be attributable to inter-population heterogeneity in genetic backgrounds, study designs, phenotype definitions, analytical methodologies, unaccounted environmental exposures or lifestyle presences^28–30^. In addition to determining the key reasons for inconsistent replications, given the distinct genetic heterogeneity and epidemiologic characteristics, it is highly suggested to construct a database of potential determinants of GDM in each racial or ethnic population.

To derive a relatively reliable estimate, we resorted to a big database from ten “AAA” hospitals in Xiamen, involving 258,466 gestational women between 2008 and 2018, and thereof data from 187,432 gestational women with pre-pregnancy diabetes mellitus were finally analyzed. To control for confounders, we adopted a graded adjustment method, and only factors that were consistently associated with the significant risk of GDM were identified. After removing factors with strong evidence of correlation, we identified eight significant factors independently associated with GDM, and six of them are laboratory biomarkers. Consistent with the results of most previous studies^16,31–34^, we here confirmed the contribution of pre-pregnancy obesity to the increased risk of having GDM, as well as the beneficial impact of pre-pregnancy intake of folic acid. Although the six laboratory biomarkers of significance identified in this study are routinely measured in clinical practice, their association with GDM is the subject of debate due to conflicting data or is rarely reported. Taking albumin as an example, Piuri and colleagues observed a significantly higher level of albumin in women with GDM than the general population^35^, whereas there was no material difference in albumin in the study by Gungor and colleague^36^. By contrast, we found that albumin level was significantly lower in women with GDM than women without GDM. A real finding can fail to replicate due to numerous reasons, including divergent genetic backgrounds and insufficient statistical power. Nevertheless, it is widely recognized that the risk attributable to a single index or biomarker is small, considering that GDM is a multifactorial disease to which inherited, environmental, and lifestyle factors contribute independently or interactively^37,38^, and such a small effect may also be exacerbated by the presence of other factors. For practical reasons, to construct a multivariable prediction model with decent prediction performance for GDM is imperative.

To shed some light on this issue, in an attempt to test prediction accuracy and justify gained benefits of eight significant factors identified in this study, we employed multiple statistics from both calibration and discrimination aspects^39^ and visual tools in decision curve analysis. On the basis of decent prediction performance, we regressed age and eight significant factors in a nomogram model, and found that this model had an 87% prediction accuracy. The importance of this nomogram prediction model lies in the facilitation of clinical appraisal of future developing GDM during the third trimester of pregnancy. For further practical application, we agree the results of the present population in Xiamen will require further validation in an independent Chinese population and additional follow-up for confirmation of this nomogram risk prediction model presented here.

Our study findings have important public health implications. In clinical practice, the diagnosis of GDM is made during the 24th to 28th weeks of pregnancy. If we can predict the later occurrence of GDM by using pre-pregnancy or early-pregnancy markers, the time window of adverse gestational consequences can be dramatically improved by immediate intervention on the high-risk pregnant women. It is worth noting that the nomogram prediction model we established can tease out 87% of women who will progress to GDM later.

### Limitations

There are some limitations to the present analysis. First, all gestational women were exclusively enrolled from ten “AAA” hospitals in Xiamen, China, and the extrapolation of our findings to the other regions or racial groups is limited. Second, other important factors such as the previous histories of GDM and macrosomia^40^, as well as sleep quality^41^ and ambient air pollution exposure^42^, which have been reported to be strong predictors for future GDM, were not available for us. Third, all laboratory biomarkers were measured only once, and it is of great interest to monitor their dynamic changes in susceptibility to the later development GDM. Fourth, most demographic data in this cohort, especially pre-pregnancy intake of folic acid, were self-reported and error-prone, and so the possibility of measurement error and residual confounding remains.

### Conclusions

Taken together, though a big data analysis, we have identified eight independent pre-/early-pregnancy predictors in significant association with the later risk of GDM, and importantly a nomogram modeling these predictors has over 85% accuracy in early detecting pregnant women who will progress to GDM at the third trimester. For practical reasons, we hope the current investigation will not remain just an endpoint instead of a start to establish background data to further explore potential risk profiling of GDM, as well as to decipher underlying molecular mechanisms.

### Ethical approval

The conduct of this study was in accordance with the ethical standards of the Institutional Review Board of each participating hospital and with the 1964 Helsinki declaration and its later amendments or comparable ethical standards.

### Informed consent

Informed consent was obtained from all study participants.

## Supplementary Information

Supplementary Informations.

## Data Availability

Data are available upon reasonable request.
