# Supplementary material for: Association of pre- and early-pregnancy factors with the risk for gestational diabetes mellitus in a large Chinese population
Source: Sci Rep. 2021 Apr 1;11:7335. doi: 10.1038/s41598-021-86818-7 (PMC8016847; doi:10.1038/s41598-021-86818-7)

Supplementary Table 1. Assessment of non-random measurement error for clinical biomarkers across the ten “AAA” hospitals.

**Clinical Biomarkers**	**ICC (confidence limit)**
Hemoglobin (g/L)	0.03 (0.01 to 0.05)
White cell count (10^9/L)	0.005 (0.00 to 0.01)
Platelet count (10^9/L)	0.03 (0.01 to 0.04)
Alanine transaminase (U/L)	0.02 (0.00 to 0.05)
Aspartate aminotransferase (U/L)	0.04 (0.02 to 0.06)
Albumin (g/L)	0.07 (0.04 to 0.10)
Direct bilirubin (μmol/L)	0.05 (0.00 to 0.09)
Creatinine (μmol/L)	0.02 (0.01 to 0.04)
Blood urea nitrogen (mmol/L)	0.06 (0.02 to 0.10)
OGTT fasting glucose (mmol/L)	0.01 (0.00 to 0.02)
OGTT1h glucose (mmol/L)	0.01 (0.00 to 0.02)
OGTT2h glucose (mmol/L)	0.02 (0.00 to 0.03)

Abbreviations: OGTT, oral glucose tolerance test; ICC, intraclass correlation coefficient.

Supplementary Figure 1. The calibration curve for the nomogram model.

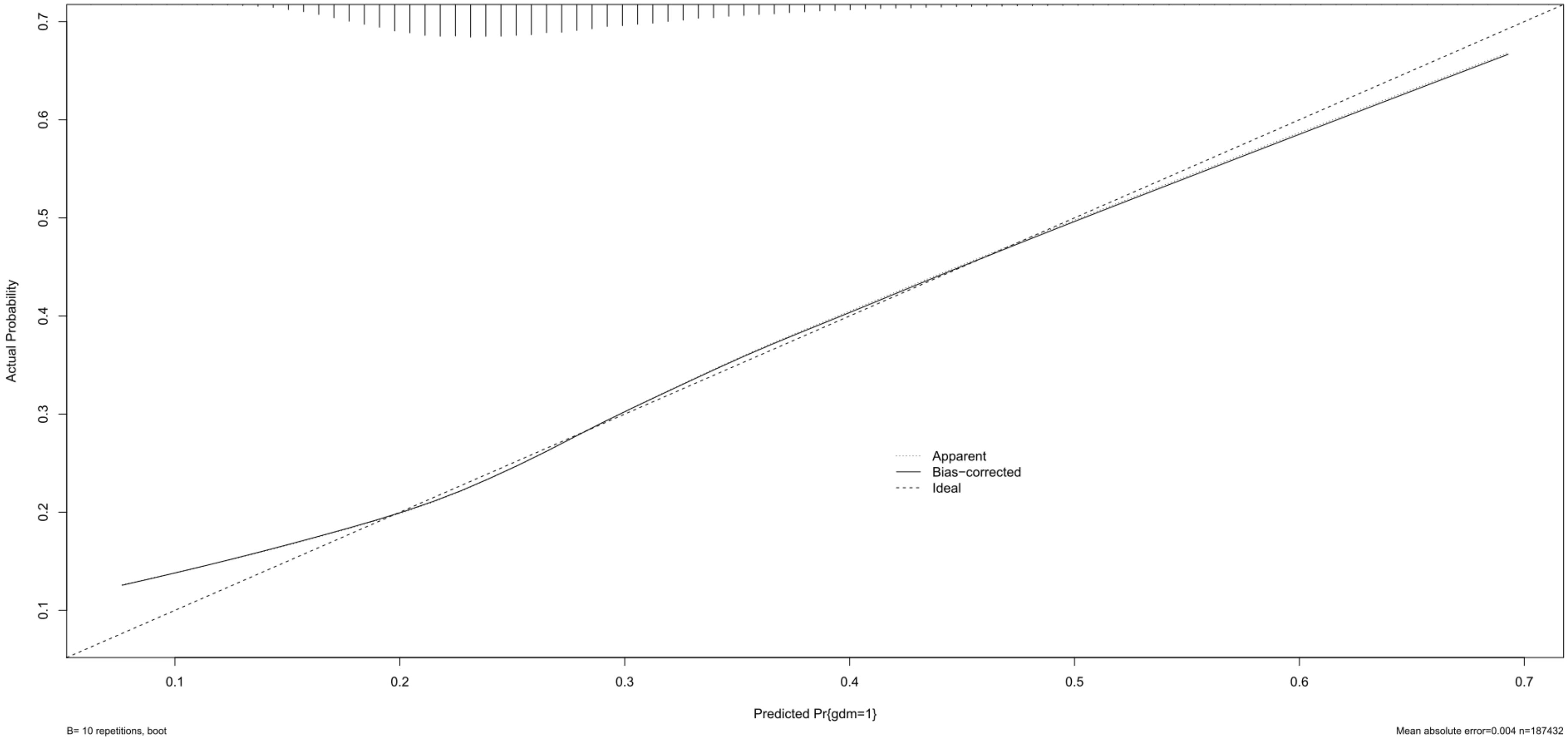

Supplement: Supplementary file 1 — Supplementary Informations. [file 41598_2021_86818_MOESM1_ESM.pdf]
